# The D. M. C’s Reply to “One of Her Boys”

**Published:** 1859-12

**Authors:** Albany Packham

**Affiliations:** D. M. C.


### THE D. M. C's. REPLY TO "ONE OF HER BOYS."

You wish to know all about the D. M. C., do you? Well, knowledge is power, and you wish to be powerful! You felt bad, did you, to see your Alma Mater humbled by one of her officers asking aid to reach Niagara by a contribution? You must have a large quantity of the milk of human kindness, to feel bad under such circumstances, so come, let me milk you a little. But in the first place, I refer you to No. 4, Dental Reporter, published by J. T. Toland, Cincinnati, O.

You confess your ignorance, eh? Then there is hope for you yet, as confession is good for the soul. No wonder you are ignorant, for you don't take the *papers*; now that is very wrong, for there is nothing like printer's ink, before breakfast and after supper—or you never would have made such an *ass* of yourself. Balaam's ass had sense and long ears, but you had the long ears, without the sense. If you are too poor to take the *papers*, I will send you either the Scientific Artisan, Scientific American, Dental Register, New York Sun, Ledger, Cincinnati Commercial, Actor, or London Times, for either one or the other have articles from the pen of your despised *baggage man*. Your slur about the aid to reach Niagara by a contribution was unprofessional and uncalled for; it was contemptible and mean, to say the least. When you ask and receive your fee for filling teeth with amalgam, do you note that down as aid, given you by contribution? not you!

I was seeking after knowledge under difficulties. Was it any disgrace in me to work for the money before I paid my expenses? was it any disgrace in me to listen all day to the talk of the *wise men*, in the Convention, and then, like Ben Franklin, the poor printer, walk the streets and eat my penny loaf? was it any disgrace to that nation, who called Cincinnatus from his plow to rule over them? Bloomfield and Burns were plowmen, Arkwright was a blacksmith, Henry Kirk White was a butcher-boy, yet all were bright, particular stars; but you (with your *hot-house, forced culture,*) will never emit a spark of sufficient brightness to see your own folly, but forever remain, what you call yourself, "one of the boys." Therein lies *your* humility.

> You would like to know who I am, would you?
> Curiosity in a woman is a curse,
> But in a man, it's ten times worse.

I am what I am; yet I will tell you somewhat of my past life (as you did not take the papers), and mark the contrasts.

I have dined in the palace of kings and queens (at Brighton Palace, England), and supped in the hut of the Indian (Texas);

I have listened to the greatest orators of their time (Lords Palmerston and Russell, Clay and Calhoun, Lamartine and Everett), and heard the vilest language ever uttered by the vilest of women (in a watch-house in New York); I have tossed upon the mighty waves of the ocean, and laid me down on the smooth prairies of New Mexico; I have soared high up into the heavens (in a balloon), and descended down into the bowels of the earth, in lead mines; I have hired servants to wait upon me, and have hired myself out to wait upon others.

I have dabbled among the Bears and Bulls, and been a "lame duck." I have seen men shot, hung, stabbed, drowned, poisoned, and blown to pieces by gas and gunpowder, yet I ran away from flying bullets; you may say that was cowardly, but I say it was wise,

> "For he that fights and runs away,
> Will live to fight another day."

I have poured two thousand bright gold dollars into my wife's lap, and now I am not worth one; yet I am rich, in fact, very wealthy, for I have one of the best of wives, and four sweet *cherry bibs*, which are worth more than all the wealth of the world to me.

You inquire what the D. M. C. means? The title was conferred upon me by those who had the power invested in them to create it. Some say it means Demonstrator of Manual Combustion, because I "fire up" before going to hear lectures; some say it means Demonstrator of Mechanical Caloric, because I was seen inside of Captain Ericson's engine, trying to discover the water supply pipe; Prof. C.; of our college, says it means Decent Man's Companion; but no doubt you think it means D——n Mean Cus. If you do, it is merely the echo from your own hollow heart.

You whine and say you felt bad. I hope you feel better now; but you may snivel and turn up your nose still higher than it naturally was, when *swaged from the male and female dies*, for you are vain enough to think it makes you look *pug-*

nacious, without your being so. Your Alma Mater was humbled, was it, because a supposed baggage man occupied one of her honorable chairs? Be easy, my boy, for that same man sat in the same identical chair that King John did when he signed the celebrated Magna Charta at Runnymede—so honor the breeches.

Having an eye to business, I would inform you that after the College closes in the spring, I mean to outdo Blondin, by going over the Falls of Niagara in a skiff, having a parachute fastened to my body, to sustain me in the air, while the skiff is dashed to atoms in the boiling waters under my feet. Price 25 cents admission to Goat Island. If you have courage enough, come along, for there's room for one more. If we survive, you shall share half of the glory; but if we perish, there will be an end of "one of the boys," as well as of the despised "baggage man."

ALBANY PACKHAM, D. M. C.

